# 2,6-Dibromo-4-butyl­aniline

**DOI:** 10.1107/S1600536810047410

**Published:** 2010-11-24

**Authors:** Liang Zhao, Li-Ping Feng

**Affiliations:** a Department of Chemical & Environmental Engineering, Anyang Institute of Technology, Anyang 455000, People’s Republic of China

## Abstract

In the title compound, C_10_H_13_Br_2_N, the amino N atom is essentially coplanar with the benzene ring, with an r.m.s. deviation of 0.004 Å. Weak intra­molecular N—H⋯Br hydrogen bonds occur. In the crystal, mol­ecules are linked into a zigzag chain parallel to the *b* axis by weak N—H⋯N hydrogen bonds.

## Related literature

For related compounds, see: Fender *et al.* (2002 ▶); Grabowski (2005 ▶); Kryatova *et al.* (2004 ▶); Lehn (1995 ▶); Pedersen (1967 ▶); Scheiner (1997 ▶).
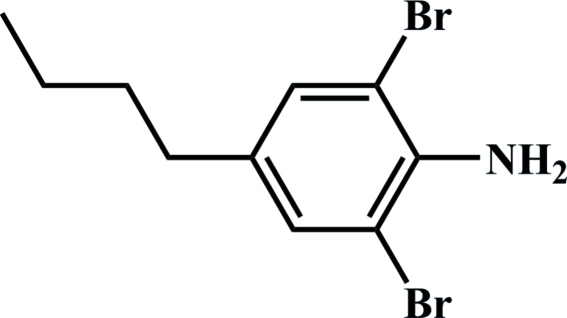


## Experimental

### 

#### Crystal data

C_10_H_13_Br_2_N
*M* *_r_* = 307.03
Monoclinic, 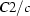

*a* = 17.566 (4) Å
*b* = 4.6083 (9) Å
*c* = 29.023 (6) Å
β = 98.93 (3)°
*V* = 2320.8 (8) Å^3^
*Z* = 8
Mo *K*α radiation
μ = 6.94 mm^−1^
*T* = 298 K
0.10 × 0.03 × 0.03 mm

#### Data collection

Rigaku Mercury2 diffractometer
Absorption correction: multi-scan (*CrystalClear*; Rigaku, 2005 ▶) *T*_min_ = 0.910, *T*_max_ = 1.000
9142 measured reflections
2633 independent reflections
1286 reflections with *I* > 2σ(*I*)
*R*_int_ = 0.117

#### Refinement

*R*[*F* ^2^ > 2σ(*F* ^2^)] = 0.069
*wR*(*F* ^2^) = 0.187
*S* = 0.98
2633 reflections
118 parameters
H-atom parameters constrained
Δρ_max_ = 0.80 e Å^−3^
Δρ_min_ = −0.60 e Å^−3^

### 

Data collection: *CrystalClear* (Rigaku, 2005 ▶); cell refinement: *CrystalClear*; data reduction: *CrystalClear*; program(s) used to solve structure: *SHELXS97* (Sheldrick, 2008 ▶); program(s) used to refine structure: *SHELXL97* (Sheldrick, 2008 ▶); molecular graphics: *SHELXTL* (Sheldrick, 2008 ▶) and *PLATON* (Spek, 2009 ▶); software used to prepare material for publication: *SHELXTL*.

## Supplementary Material

Crystal structure: contains datablocks I, global. DOI: 10.1107/S1600536810047410/dn2624sup1.cif

Structure factors: contains datablocks I. DOI: 10.1107/S1600536810047410/dn2624Isup2.hkl

Additional supplementary materials:  crystallographic information; 3D view; checkCIF report

## Figures and Tables

**Table 1:** Hydrogen-bond geometry (Å, °)

*D*—H⋯*A*	*D*—H	H⋯*A*	*D*⋯*A*	*D*—H⋯*A*
N1—H1*A*⋯N1^i^	0.86	2.53	3.181 (7)	134
N1—H1*A*⋯Br1	0.86	2.68	3.095 (5)	111
N1—H1*B*⋯Br2	0.86	2.64	3.074 (5)	113

Symmetry code: (i) 
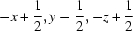
.

**Fig. 1.**
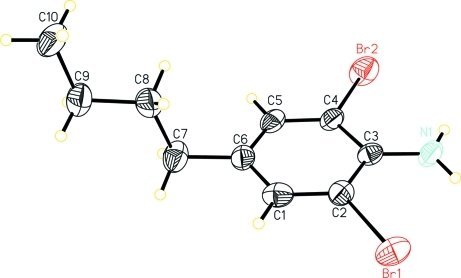
A view of the title compound with the atomic numbering scheme. Displacement ellipsoids are drawn at the 30% probability level. H atoms are represented as small spheres of arbitrary radii.

**Fig. 2.**
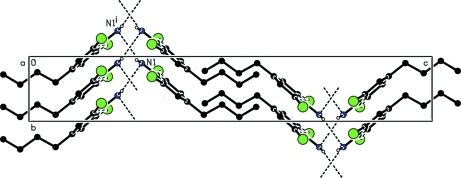
Partial packing view of the title compound along the a axis. H atoms not involved in hydrogen bonding (dashed lines) have been omitted for clarity. [Symmetry code: (i) -x+1/2, y-1/2, -z+1/2]

## supplementary crystallographic information

### Comment

In recent years there has been a rapidly increasing interest in the construction of various kinds of supramolecular systems for understanding molecular self-assembly principles and for designing molecular recognition devices (Fender *et al.*, 2002; Kryatova *et al.*, 2004; Pedersen, 1967). The supramolecular system generally refers to an assembly of molecules which are not covalently connected but assembled by other weak intermolecular interactions, such as hydrogen bonds (Grabowski, 2005; Lehn, 1995; Scheiner, 1997). We report here the crystal structure of the title compound, 2,6-dibromo-4-butylaniline.

In the title compound (Fig.1), the N atom of the amine group is essentially coplanar with the phenyl ring, with a r.m.s. deviation of 0.004 Å. This planar conformation might be resulting from weak intramolecular N-H···Br hydrogen bonds (Table 1). The butyl group is twisted with respect to the phenyl ring resulting in torsion angles of -179.1 (7)° for C9—C8—C7—C6 and -174.7 (7)° for C7—C8—C9—C10. Bond lengths and angles lie within normal ranges.

In the crystal structure, the organic molecules are linked to form a one-dimensional chain along *b* axis by N1—H···N1 hydrogen bonds (Table 1, Fig.2).

### Experimental

2,6-dibromo-4-butylaniline (3 mmol) was dissolved in ethanol (20 ml). The solution was allowed to evaporate to obtain colourless block-shaped crystals of the title compound.

### Refinement

All H atoms attached to C and N atoms were calculated geometrically and treated as riding on their parent atoms with C–H = 0.93 Å (aromatic), 0.96 Å (methyl),  0.97 Å (methylene) and N-H = 0.86 Å, with *U*_iso_(H) = 1.2Ueq(C, N) or *U*_iso_(H) = 1.5Ueq(C_methyl_).

### Crystal data

**Table:**

C_10_H_13_Br_2_N	*F*(000) = 1200
*M**_r_* = 307.03	*D*_x_ = 1.757 Mg m^−^^3^
Monoclinic, *C*2/*c*	Mo *K*α radiation, λ = 0.71073 Å
Hall symbol: -C 2yc	Cell parameters from 2633 reflections
*a* = 17.566 (4) Å	θ = 3.4–27.5°
*b* = 4.6083 (9) Å	µ = 6.94 mm^−^^1^
*c* = 29.023 (6) Å	*T* = 298 K
β = 98.93 (3)°	Block, colourless
*V* = 2320.8 (8) Å^3^	0.10 × 0.03 × 0.03 mm
*Z* = 8	

### Data collection

**Table:**

Rigaku Mercury2 diffractometer	2633 independent reflections
Radiation source: fine-focus sealed tube	1286 reflections with *I* > 2σ(*I*)
graphite	*R*_int_ = 0.117
Detector resolution: 13.6612 pixels mm^-1^	θ_max_ = 27.5°, θ_min_ = 3.4°
CCD profile fitting scans	*h* = −22→22
Absorption correction: multi-scan (*CrystalClear*; Rigaku, 2005)	*k* = −5→5
*T*_min_ = 0.910, *T*_max_ = 1.000	*l* = −37→37
9142 measured reflections	

### Refinement

**Table:**

Refinement on *F*^2^	Primary atom site location: structure-invariant direct methods
Least-squares matrix: full	Secondary atom site location: difference Fourier map
*R*[*F*^2^ > 2σ(*F*^2^)] = 0.069	Hydrogen site location: inferred from neighbouring sites
*wR*(*F*^2^) = 0.187	H-atom parameters constrained
*S* = 0.98	*w* = 1/[σ^2^(*F*_o_^2^) + (0.063*P*)^2^] where *P* = (*F*_o_^2^ + 2*F*_c_^2^)/3
2633 reflections	(Δ/σ)_max_ < 0.001
118 parameters	Δρ_max_ = 0.80 e Å^−^^3^
0 restraints	Δρ_min_ = −0.60 e Å^−^^3^

### Special details

**Table:**

Geometry. All esds (except the esd in the dihedral angle between two l.s. planes) are estimated using the full covariance matrix. The cell esds are taken into account individually in the estimation of esds in distances, angles and torsion angles; correlations between esds in cell parameters are only used when they are defined by crystal symmetry. An approximate (isotropic) treatment of cell esds is used for estimating esds involving l.s. planes.
Refinement. Refinement of F^2^ against ALL reflections. The weighted R-factor wR and goodness of fit S are based on F^2^, conventional R-factors R are based on F, with F set to zero for negative F^2^. The threshold expression of F^2^ > 2sigma(F^2^) is used only for calculating R-factors(gt) etc. and is not relevant to the choice of reflections for refinement. R-factors based on F^2^ are statistically about twice as large as those based on F, and R- factors based on ALL data will be even larger.

### Fractional atomic coordinates and isotropic or equivalent isotropic displacement parameters (Å^2^)

**Table:**

	*x*	*y*	*z*	*U*_iso_*/*U*_eq_	
Br1	0.14631 (4)	0.22239 (18)	0.32043 (3)	0.0773 (4)	
Br2	0.46385 (4)	0.3101 (2)	0.30522 (3)	0.0848 (4)	
C3	0.3061 (3)	0.2877 (13)	0.31725 (18)	0.0482 (17)	
C6	0.3272 (5)	0.6955 (13)	0.3926 (2)	0.0576 (18)	
C2	0.2466 (3)	0.3776 (13)	0.3404 (2)	0.0512 (16)	
C4	0.3781 (3)	0.4182 (15)	0.33358 (18)	0.0533 (16)	
C5	0.3878 (4)	0.6137 (13)	0.36996 (19)	0.0544 (17)	
H5	0.4363	0.6931	0.3796	0.065*	
C1	0.2562 (4)	0.5724 (16)	0.3769 (2)	0.0596 (18)	
H1	0.2141	0.6218	0.3912	0.072*	
N1	0.2963 (3)	0.1017 (12)	0.28006 (16)	0.0621 (15)	
H1A	0.2513	0.0335	0.2698	0.074*	
H1B	0.3351	0.0530	0.2670	0.074*	
C8	0.3747 (5)	0.7421 (14)	0.4792 (2)	0.076 (2)	
H8A	0.3404	0.5851	0.4847	0.091*	
H8B	0.4235	0.6566	0.4749	0.091*	
C7	0.3402 (4)	0.8986 (16)	0.43391 (19)	0.072 (2)	
H7A	0.2914	0.9862	0.4380	0.086*	
H7B	0.3747	1.0530	0.4277	0.086*	
C9	0.3877 (4)	0.9328 (19)	0.5218 (2)	0.086 (2)	
H9A	0.4182	1.0995	0.5155	0.104*	
H9B	0.3384	1.0034	0.5282	0.104*	
C10	0.4278 (5)	0.7778 (17)	0.5637 (2)	0.102 (3)	
H10A	0.4352	0.9084	0.5898	0.153*	
H10B	0.4769	0.7094	0.5577	0.153*	
H10C	0.3971	0.6160	0.5707	0.153*	

### Atomic displacement parameters (Å^2^)

**Table:**

	*U*^11^	*U*^22^	*U*^33^	*U*^12^	*U*^13^	*U*^23^
Br1	0.0492 (5)	0.0947 (7)	0.0848 (6)	−0.0081 (4)	0.0005 (4)	0.0069 (4)
Br2	0.0537 (6)	0.1252 (9)	0.0770 (6)	−0.0047 (4)	0.0148 (4)	−0.0111 (4)
C3	0.046 (4)	0.059 (4)	0.039 (3)	0.001 (3)	0.004 (3)	0.008 (3)
C6	0.080 (5)	0.046 (4)	0.043 (4)	0.008 (4)	−0.004 (3)	0.005 (3)
C2	0.055 (4)	0.045 (4)	0.052 (4)	−0.004 (3)	0.002 (3)	0.008 (3)
C4	0.047 (4)	0.066 (5)	0.044 (3)	0.001 (3)	−0.003 (3)	0.000 (3)
C5	0.058 (4)	0.057 (4)	0.044 (4)	−0.004 (3)	−0.004 (3)	0.010 (3)
C1	0.054 (5)	0.076 (5)	0.051 (4)	0.011 (4)	0.014 (3)	0.017 (4)
N1	0.057 (3)	0.075 (4)	0.049 (3)	−0.005 (3)	−0.008 (2)	−0.008 (3)
C8	0.094 (6)	0.073 (5)	0.059 (4)	0.018 (4)	0.010 (4)	−0.004 (4)
C7	0.099 (6)	0.062 (5)	0.051 (4)	−0.009 (4)	0.002 (4)	0.000 (4)
C9	0.112 (6)	0.091 (6)	0.054 (4)	0.011 (5)	0.005 (4)	−0.014 (4)
C10	0.104 (7)	0.146 (9)	0.050 (4)	0.024 (5)	−0.008 (4)	−0.009 (5)

### Geometric parameters (Å, °)

**Table:**

Br1—C2	1.906 (6)	N1—H1B	0.8600
Br2—C4	1.891 (6)	C8—C9	1.504 (9)
C3—N1	1.368 (7)	C8—C7	1.539 (9)
C3—C2	1.392 (8)	C8—H8A	0.9700
C3—C4	1.414 (8)	C8—H8B	0.9700
C6—C1	1.382 (9)	C7—H7A	0.9700
C6—C5	1.387 (9)	C7—H7B	0.9700
C6—C7	1.510 (9)	C9—C10	1.491 (10)
C2—C1	1.379 (8)	C9—H9A	0.9700
C4—C5	1.378 (8)	C9—H9B	0.9700
C5—H5	0.9300	C10—H10A	0.9600
C1—H1	0.9300	C10—H10B	0.9600
N1—H1A	0.8600	C10—H10C	0.9600
			
N1—C3—C2	123.7 (5)	C7—C8—H8A	108.6
N1—C3—C4	121.9 (5)	C9—C8—H8B	108.6
C2—C3—C4	114.3 (5)	C7—C8—H8B	108.6
C1—C6—C5	116.9 (6)	H8A—C8—H8B	107.6
C1—C6—C7	122.2 (7)	C6—C7—C8	112.2 (6)
C5—C6—C7	120.9 (6)	C6—C7—H7A	109.2
C1—C2—C3	123.6 (6)	C8—C7—H7A	109.2
C1—C2—Br1	118.3 (5)	C6—C7—H7B	109.2
C3—C2—Br1	118.0 (5)	C8—C7—H7B	109.2
C5—C4—C3	122.1 (6)	H7A—C7—H7B	107.9
C5—C4—Br2	119.6 (5)	C10—C9—C8	112.6 (7)
C3—C4—Br2	118.2 (4)	C10—C9—H9A	109.1
C4—C5—C6	121.9 (6)	C8—C9—H9A	109.1
C4—C5—H5	119.0	C10—C9—H9B	109.1
C6—C5—H5	119.0	C8—C9—H9B	109.1
C2—C1—C6	121.1 (6)	H9A—C9—H9B	107.8
C2—C1—H1	119.4	C9—C10—H10A	109.5
C6—C1—H1	119.4	C9—C10—H10B	109.5
C3—N1—H1A	120.0	H10A—C10—H10B	109.5
C3—N1—H1B	120.0	C9—C10—H10C	109.5
H1A—N1—H1B	120.0	H10A—C10—H10C	109.5
C9—C8—C7	114.7 (6)	H10B—C10—H10C	109.5
C9—C8—H8A	108.6		
			
N1—C3—C2—C1	−177.8 (6)	C1—C6—C5—C4	−0.2 (9)
C4—C3—C2—C1	−1.3 (8)	C7—C6—C5—C4	176.9 (6)
N1—C3—C2—Br1	2.2 (8)	C3—C2—C1—C6	0.8 (10)
C4—C3—C2—Br1	178.7 (4)	Br1—C2—C1—C6	−179.1 (5)
N1—C3—C4—C5	177.6 (5)	C5—C6—C1—C2	0.0 (9)
C2—C3—C4—C5	1.1 (8)	C7—C6—C1—C2	−177.1 (6)
N1—C3—C4—Br2	−4.1 (8)	C1—C6—C7—C8	97.6 (8)
C2—C3—C4—Br2	179.3 (4)	C5—C6—C7—C8	−79.4 (8)
C3—C4—C5—C6	−0.3 (9)	C9—C8—C7—C6	−179.1 (6)
Br2—C4—C5—C6	−178.6 (4)	C7—C8—C9—C10	−174.7 (7)

### Hydrogen-bond geometry (Å, °)

**Table:**

*D*—H···*A*	*D*—H	H···*A*	*D*···*A*	*D*—H···*A*
N1—H1A···N1^i^	0.86	2.53	3.181 (7)	134
N1—H1A···Br1	0.86	2.68	3.095 (5)	111
N1—H1B···Br2	0.86	2.64	3.074 (5)	113

Symmetry codes: (i) −*x*+1/2, *y*−1/2, −*z*+1/2.
